# Towards Web-based representation and processing of health information

**DOI:** 10.1186/1476-072X-8-3

**Published:** 2009-01-21

**Authors:** Sheng Gao, Darka Mioc, Xiaolun Yi, Francois Anton, Eddie Oldfield, David J Coleman

**Affiliations:** 1 Department of Geodesy and Geomatics Engineering, University of New Brunswick, Fredericton, New Brunswick, Canada; 2 Service New Brunswick, Fredericton, New Brunswick, Canada; 3 Department of Informatics and Mathematical Modelling, Technical University of Denmark, Denmark; 4 New Brunswick Lung Association, Fredericton, New Brunswick, Canada

## Abstract

**Background:**

There is great concern within health surveillance, on how to grapple with environmental degradation, rapid urbanization, population mobility and growth. The Internet has emerged as an efficient way to share health information, enabling users to access and understand data at their fingertips. Increasingly complex problems in the health field require increasingly sophisticated computer software, distributed computing power, and standardized data sharing. To address this need, Web-based mapping is now emerging as an important tool to enable health practitioners, policy makers, and the public to understand spatial health risks, population health trends and vulnerabilities. Today several web-based health applications generate dynamic maps; however, for people to fully interpret the maps they need data source description and the method used in the data analysis or statistical modeling. For the representation of health information through Web-mapping applications, there still lacks a standard format to accommodate all fixed (such as location) and variable (such as age, gender, health outcome, etc) indicators in the representation of health information. Furthermore, net-centric computing has not been adequately applied to support flexible health data processing and mapping online.

**Results:**

The authors of this study designed a HEalth Representation XML (HERXML) schema that consists of the semantic (e.g., health activity description, the data sources description, the statistical methodology used for analysis), geometric, and cartographical representations of health data. A case study has been carried on the development of web application and services within the Canadian Geospatial Data Infrastructure (CGDI) framework for community health programs of the New Brunswick Lung Association. This study facilitated the online processing, mapping and sharing of health information, with the use of HERXML and Open Geospatial Consortium (OGC) services. It brought a new solution in better health data representation and initial exploration of the Web-based processing of health information.

**Conclusion:**

The designed HERXML has been proven to be an appropriate solution in supporting the Web representation of health information. It can be used by health practitioners, policy makers, and the public in disease etiology, health planning, health resource management, health promotion and health education. The utilization of Web-based processing services in this study provides a flexible way for users to select and use certain processing functions for health data processing and mapping via the Web. This research provides easy access to geospatial and health data in understanding the trends of diseases, and promotes the growth and enrichment of the CGDI in the public health sector.

## Background

Population growth, rapid urbanization, environmental degradation, and the misuse of antimicrobials have disrupted the equilibrium of the microbial world, causing the rise of new emerging diseases [1]. Health information is very useful in helping people to understand health phenomena, mitigate disease outbreaks, and analyze disease etiology. However, most public health departments typically collect data as needed and maintain it locally, and this unavoidably limits the access to important public health data for health researchers and the public [2]. World Health Organization [1] pointed out that keeping disease outbreaks secret is no longer feasible and sharing essential health information is one of the most feasible routes to global public health security. Sharing health information through the Web provides flexible and real-time data access, and assists people to discover and use this information. Currently, many health departments have begun to provide public access to their health statistics via the Internet, and this promotes interest in user involvement and data-set exploration [3]. Some health information like morbidity and mortality indicators has become obtainable to health professionals and the public by means of the Internet [4]. With the new updated health cases collected from hospitals or surveys, the Web can distribute this information to users in real time. Distributing and sharing health information via the Web can assist authorities and decision makers across health jurisdictions to collaborate in preventing, controlling, and responding to a specific disease outbreak at both the local and national levels. Current Web 2.0 technologies can further facilitate data sharing and collaboration between users, and the Web 2.0 mash-up allows the combination of multiple third-party services over the Web [5,6]. An example of a mash-up is the combination of bird flu case data with Google maps to visualize the distribution of disease for health surveillance.

Health information is collected through two kinds of georeferences. One kind is the point data which record the coordinates of disease case location. The other kind is region data which is collected as a summary for a geographical area. To represent health information, especially over the Web, the privacy and confidentiality concerns are given a lot of thought. Laws governing use and distribution of public health information should be respected in each jurisdiction, and yet the need for information to support critical decision making on public health threats like Tuberculosis, Avian Flu, and Influenza should be met. To keep the privacy of health information while maintaining highly informative data, health data should be represented at the aggregate level, with high privileges to see more detailed data.

Maps are powerful tools to classify, visualize, communicate and navigate space and/or spatial relations in the data which would be hard to explore otherwise [7]. With maps, it is easy to discover adjacent neighborhood similarities as well as spatial patterns that are hidden in health data. Two kinds of Web-based maps exist: view-only maps and interactive maps [8]. The view-only maps are the cartographical representation of data in images such as GIF, PNG or JPEG format. Interactive maps can respond to some mouse actions on the map, with the technologies such as Scale Vector Graphics (SVG,) Extensible 3D (X3D) Graphics, and Virtual Reality Markup Language (VRML). Kamadjeu and Tolentino [9] discuss the advantages of use the SVG in web cartographical representation, such as smaller and more compressible files, pure XML, human readability, scalability and support from major industries.

From our previous study for health mapping, we found that the quality of health data representation in Web-based GIS applications was still limited [10]. Even though many Web-based health applications dynamically generate view-only maps or interactive maps, certain information is missing for people to fully interpret the map such as the data source description and the method used in the data aggregation process. Consideration of the source and quality of the health data can help health practitioners, the general public, and policy makers to evaluate the trustworthiness of spatial analysis results [3]. In addition, scientific users want to know details about methodology when evaluating representations. For the representation of health information to users, the following issues should be considered:

1) The metadata of the health information. The description of the health data is important in understanding data sources and quality.

2) The statistical methodologies used. The description of the statistical methods for representing health data can be used to determine the quality of the results.

3) The comprehensiveness of the representation. The representation which can combine many kinds of form of representations (text, maps, graphics, etc.) will assist people in exploring the health phenomena with less misinterpretation.

4) The consistency of the cartographical representation. Health information should be mapped in the same patterns regardless of platform or system.

5) The semantic meaning. Shared vocabularies or styles can eliminate different interpretations.

Thus, a health data representation format needs to be developed to fulfill these five requirements and enhance the sharing of health information via the Web. In the health decision-making process, usually we need to integrate health data from heterogeneous sources. With a suitable health data representation model that catches all the aspects of health information, we can more easily understand health data and integrate the health data from different sources together.

Meanwhile, Web-based processing could take advantage of net-centric and collaborative computing and let users select the processing tools flexibly [11]. In the case that local health departments are not familiar with statistical methodologies in health data processing, it may inevitably take a steep learning curve to apply the processing methodologies [12]. In addition, it is hard to build a system that includes every complex function. Web-based processing allows users to select the cost-effective processing and mapping tools to accomplish a task, without the need to purchase advanced hardware or software. However, to date Web-based processing has not been adequately utilized for flexible processing of health information.

## Methods

### XML and OGC Web Services

The sharing of health information is critical for preventing diseases, responding to emergencies, and educating the public and policy makers. However, many health professionals and authorities do not have tools to map health information in some cases they cannot visualize health information to make time-sensitive decisions, since they do not have the time, money, or skills to statistically analyze vast amounts of distributed data and render aggregated results into a geographic interface for interpretation. XML, web services, and related standards, have matured, yet confidence in such technology to visualize or share health information is only beginning to emerge.

XML, as a platform independent language, can support information interchange and representation through the Web. XML has many advantages, such as platform and application independence, extensibility, user-driven development and an open standard for data interchange via the Internet [13]. Health Level 7 (HL7) standards promote health care information exchange through XML [14]. HL7 Clinical Document Architecture (CDA) is an XML standard used to exchange clinical documents. For example, an XML document can record the information of a patient's allergy to certain medicines. But the primary domain of HL7 standards is clinical and administrative data, and explicit spatial information and health data mapping are not considered. Therefore, a standard format in sharing the representation of health information in time and space is needed.

To overcome the disadvantages of tightly coupled systems and improve their reusability, the concept of Service Oriented Architecture (SOA) has gained popularity recently. SOA provides a flexible way to share data as well as processing functions over the Internet to reduce costs of building complex systems. SOA has many benefits, such as better return on investment, better maintainability, higher availability, flexible service assembly, more security, and support for multiple client types [15]. The Open Geospatial Consortium (OGC) initiated the Open Web Service (OWS) program based on service-oriented architectures and web services (a common implementation of service oriented architectures), and has proposed several geospatial specifications to support geospatial data sharing and interoperation, such as Web Map Service (WMS), Web Feature Service (WFS), and Web Processing Service (WPS). WMS publishes its ability to produce maps rather than its ability to access specific data holdings, and generates spatially referenced maps dynamically [16]. WFS defines the interfaces for the access and manipulation of geographical features and elements through Geography Markup Language (GML) [17]. WPS provides standardized interfaces to facilitate publishing, discovering and binding geospatial services that enable spatial processing functions across a network [18]. It regulates the connection rules of input request and output response that govern the geospatial processing event. The interfaces (GetCapabilities, DescribeProcess, and Execute) define how the client and server can cooperate in the execution of a process and generate the processing results. The data used in the WPS can be stored at the server side or acquired from a network. Accessing health information through standard interfaces is important to achieve data accessing and interoperability. Using the standard geospatial service interfaces, the wide access of health information can improve the ability to intervene in health issues, inform the public of the availability of resources, reduce the number of people affected by illness, strengthen the cooperation between different health organizations, and therefore reduce costs to the health care system.

### HEalth Representation XML (HERXML)

The HEalth Representation XML (HERXML) schema is designed for the sharing of health data cartographical representation, data source description and statistical methodologies used via the Web. There are different kinds of health activities, such as hospital observation, laboratory tests and results, healthcare and medication services, and training and education for patients. Since these activities are social events and related to spatial location, the proper way to support mapping of these activities on maps is a foremost concern in geographic health applications. In the mapping of health-related activities, statistical methods can be used to connect health-related activities with maps. The methods to generate maps from health-related activities need to be considered. The following statistical methods are applied in this research: Crude Morbidity Rate (CMR), Normalized Morbidity Ratio (NMR), Age-Specific Morbidity Ratio (ASMR), Age-Adjusted Morbidity Ratio (AAMR), and Standardized Morbidity Ratio (SMR), Summation, Mean, Standard Deviation, Variance, Skewness and Kurtosis. These statistical methods consider spatial, temporal, and demographic factors and their influence on health related activities, which can show the health information distribution with spatial, temporal, age, and gender differences. Other statistical methods can be introduced to analyze other influential factors.

The intention is to make the HERXML schema able to support the Web-based representation of health information for users to interpret the statistical results. Three dimensions of representation are related with spatial data: semantic, geometric and graphical [19]. Therefore, we include these three kinds of representations in the HERXML schema. Semantic representation describes the health related activities, data sources, and the statistical methods used. Geometric dimension shows what type of geometry (point, line, and polygon) will be used to represent these health data. Graphic representation defines what styles or symbols are used to generate health maps.

**Figure 1:**
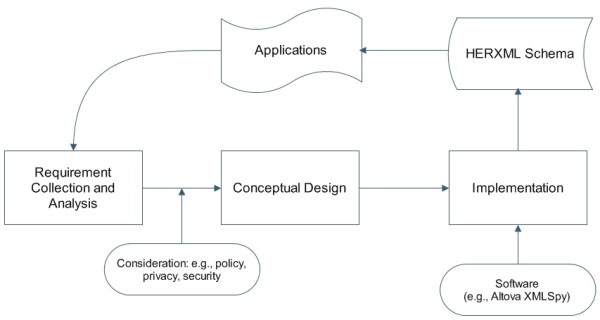
**HERXML schema design process**. The HERXML schema design process follows a cyclic development. The steps include user requirement collection and analysis, conceptual design, schema implementation, and schema validation in applications.

The design of the HERXML follows an iterative process, as shown in Figure 1. It starts with user requirement collection and analysis, such as the range health information, related influential factors, and ways of representations. With the consideration of policy, privacy and security issues, the main concepts used in the representation of health information are determined. Next, a XML design software tool, Altova XMLSpy [20] is used to encode the HERXML schema. After that, the HERXML schema will be tested in application to validate user requirements. The iteration continues with a new version of HERXML schema until the end users satisfy. With the above cyclic development process, our preliminary HERXML schema used in this project is defined (refer to Additional file 1).

**Figure 2:**
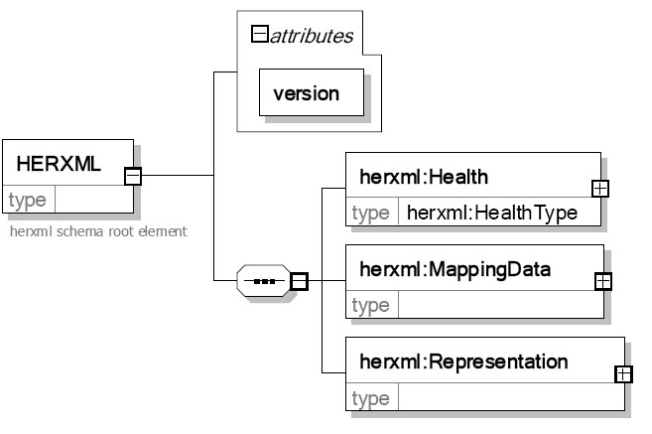
**The HERXML schema**. The HERXML includes a "Health" part, a "MappingData" part, and a "Representation" part.

As shown in Figure 2, the designed HERXML schema includes three parts: health, mapping data, and representation.

The health part includes the basic information of the health-related activities, with the name, title, description, and keyword list elements, and a type attribute. HealthType is an abstract complex type. It can be extended to support disease observation or other activities.

**Figure 3:**
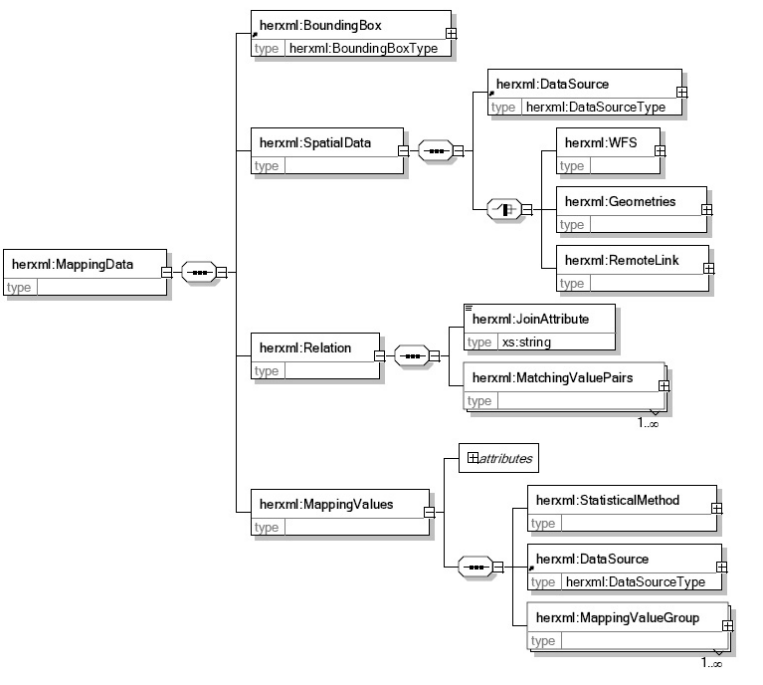
**The mapping data part schema**. The "mapping data" part schema includes a "BoundingBox" component, a "SpatialData" component, a "Relation" component and a "MappingValues" component.

The mapping data part mainly records the data used for mapping. As shown in Figure 3, it includes the bounding box of the data, the spatial data, the relation between spatial data and mapping values, and the mapping values.

• "BoundingBox" represents the spatial range of the mapping data.

• "SpatialData" could be GML from WFS services, GML records, or Xlink to GML databases. The data source item is used to show the metadata of the spatial data. The health data are statistical values and are linked with the spatial data through the joining attribute.

• "Relation" records the linking attribute and the matching ID value of both spatial data and mapping values.

• "Mapping values" includes the health data source description, the statistical method used and the mapping value lists. The statistical method part describes the name, title, description, data source, and statistical parameters of the statistical method used. The data source description shows metadata of health information, such as the source of the data, the time range of data, and the contact information. Statistical methods are used to generate classification maps and charts for health related activities. We predefined some parameters from the spatial, temporal, and demographic aspects for public health, such as AgeFrom, AgeTo, and StartTime, which can show health distributions with spatial, temporal, age and gender differences. Users can add additional parameters in the parameter group to support advanced statistical methods.

The Representation part defines the style used to represent health maps. It describes the default representation bounding box and style description. Depending on the kind of representation, the StyleType is extended to ChartStyleType, PointStyleType, LineStyleType, and PolygonStyleType. For instance, the PolygonStyleType includes the border and fill elements. The type of filling in a polygon can be gradient fill or range-based fill. For the range-based fill, the fill method can use color, pattern, and texture. The border element contains the color, line style and line weight of the border.

### WPS for health data processing with HERXML

**Figure 4:**
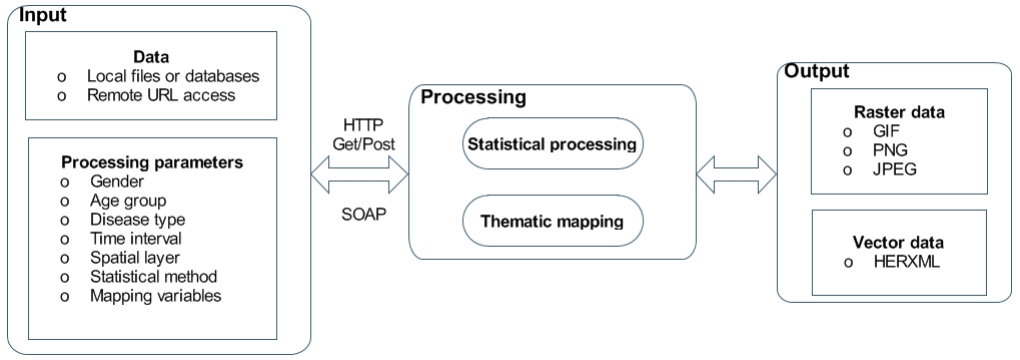
**A WPS for health data processing**. The flow shows the input data, output data, and processing components of the designed WPS.

The procedure of our WPS design is shown in Figure 4. The input includes health data and parameters. The health data for the Web-based processing could be stored in the server (in databases or files) or acquired through remote access (through web services or remote transfers). The parameters can be encoded by Key/Value pairs or XML, including the disease type, gender, age group, statistical method, time interval, spatial layer, and thematic mapping variables. The output of the processing could be either in the raster data format (JPEG, PNG, GIF) or in the vector data format (HERXML). The use of HERXML in processing can enhance people's understanding of the resulting health information mapped. In the configuration of the WPS, the access of WPS can be limited to certain domains or IP addresses. The WPS can be further divide into fine granularity, with one processing service for the statistical calculation and the other processing service for the thematic mapping.

### Architecture for health data processing and sharing

**Figure 5:**
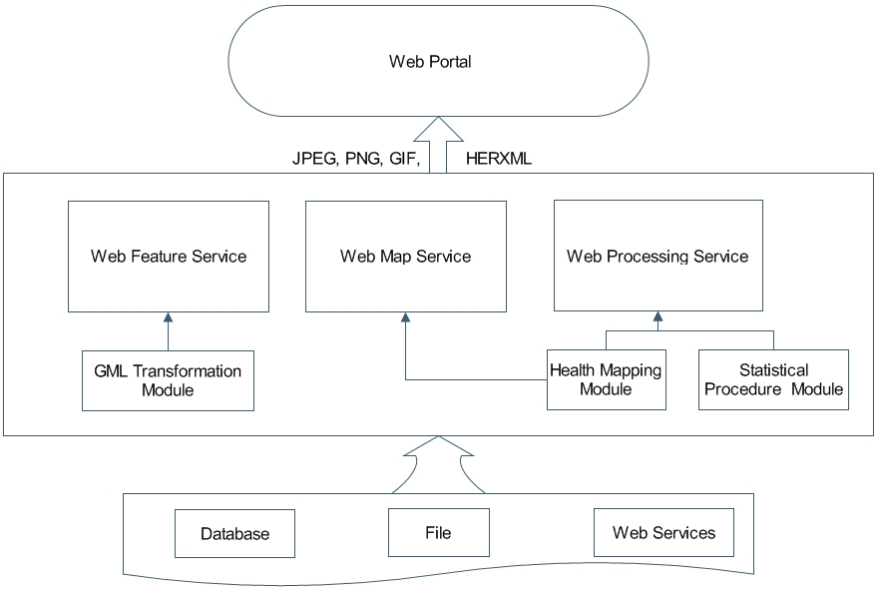
**Implemented health data processing and sharing architecture**. The architecture contains a data tier, a service tier, and a web portal tier.

To implement a Web-based application for statistical exploration of health information, service oriented architecture is an effective solution [21]. In this research, we implement the standard OGC services including WMS, WFS, and WPS. The proposed architecture (see Figure 5) includes three tiers: a data tier, a service tier, and a web portal tier.

The data tier stores all the health data and related data for health studies. These data could be available from databases or web services.

The service tier implements WMS, WFS, and WPS for health studies.

• WMS provides standard interfaces to generate maps and charts for visualization of health information. It utilizes the health mapping module to generate maps to show events or facilities distribution. The input data could be obtained from HERXML, GML, WFS, WPS, DBMS, or files.

• WFS uses the GML transformation module to share spatial data through GML. It can be linked with the mapping values (part of HERXML) to create thematic health maps.

• WPS is used to analyze spatio-temporal health data. The health data analysis supports data rolling up from a low spatial level to a high spatial level. WPS uses the health mapping module and statistical procedures. The input data of WPS could be obtained through WFS, GML, DBMS, or files.

The web portal tier is a client for the visualization of disease data and maps. It can bring together different facets of health information into one location to improve health promotion, health care research, education, and policy making.

## Results

A case study has been carried on the development of web application and services within the Canadian Geospatial Data Infrastructure (CGDI) framework for community health programs of the New Brunswick Lung Association. The Canadian Geospatial Data Infrastructure (CGDI) aims to support online access to location-based information which can efficiently help people in their decision making [22]. One priority area of CGDI is to share location-based information for analyzing and monitoring public health. Sharing of health information in the CGDI will improve our ability to intervene on health issues, and inform the public of the availability of resources.

**Figure 6:**
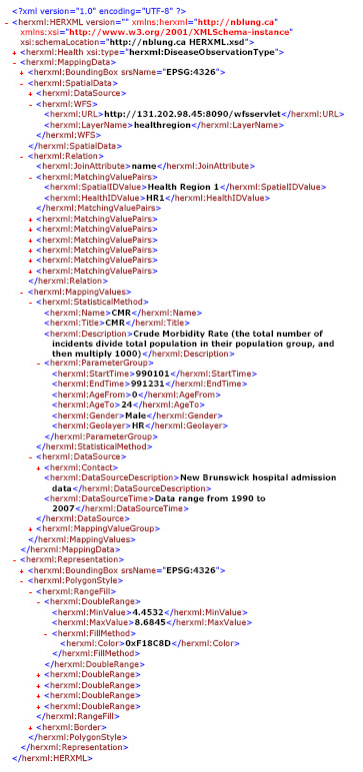
**An HERXML document generated from a WPS**. This HERXML document represents a processing result from a WPS.

**Figure 7:**
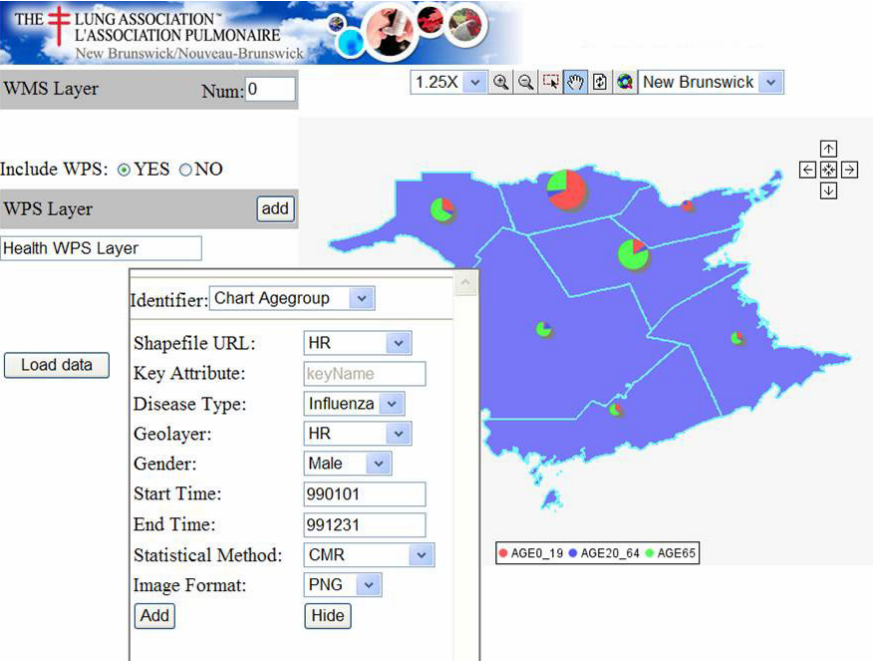
**A map generated from a WPS**. This map represents a processing result in image format from a WPS.

The health data we used in this study include four kinds of respiratory disease data (Asthma, COPD, Influenza, and Cancer) collected by the New Brunswick Lung Association. The disease data are geo-coded to spatial position through the postcode. The spatial data we used include the six levels of spatial boundary data that cover the entire territory of New Brunswick. The six levels are "Province," "Health Region," "Census Division," "Census Subdivision, " "Forward Sortation Area," and "Dissemination Area" geo-layers. All the health data and geometrical boundary data are stored in Oracle database. Low counts (i.e., less than five observations) or false counts are not represented to further ensure privacy and accuracy. WMS services are used to publish the health facility distribution maps. WFS services distribute the different levels of spatial boundary data. In this study, we provide access to new Web Processing Services in the CGDI to enable statistical representation of health information. The WPS services support the statistical calculation as well as mapping of the health data. Figure 6 shows an example of an HERXML document generated by a WPS, and Figure 7 presents a map representation generated from a WPS.

**Figure 8:**
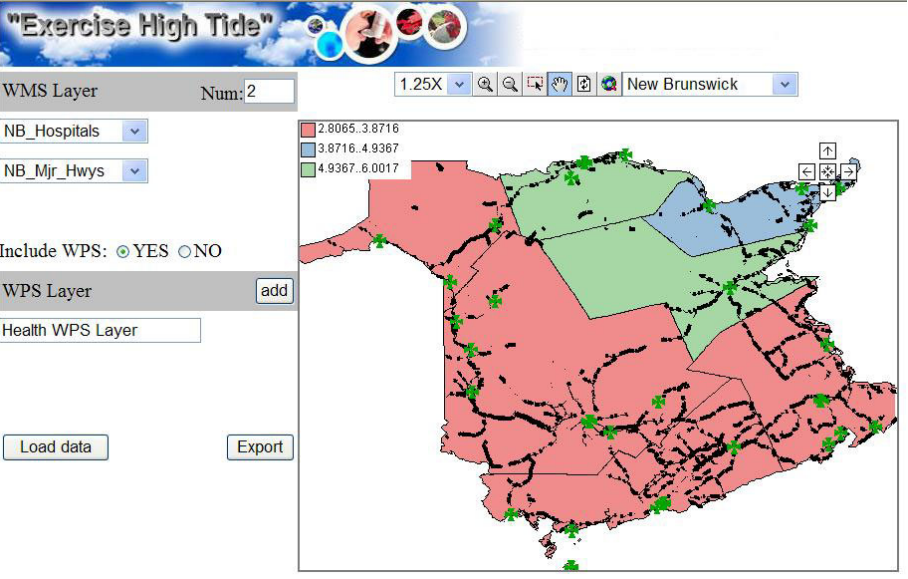
**The configuration wizard interface**. The configuration wizard manages the WMS layers and the parameters for the WPS.

**Figure 9:**
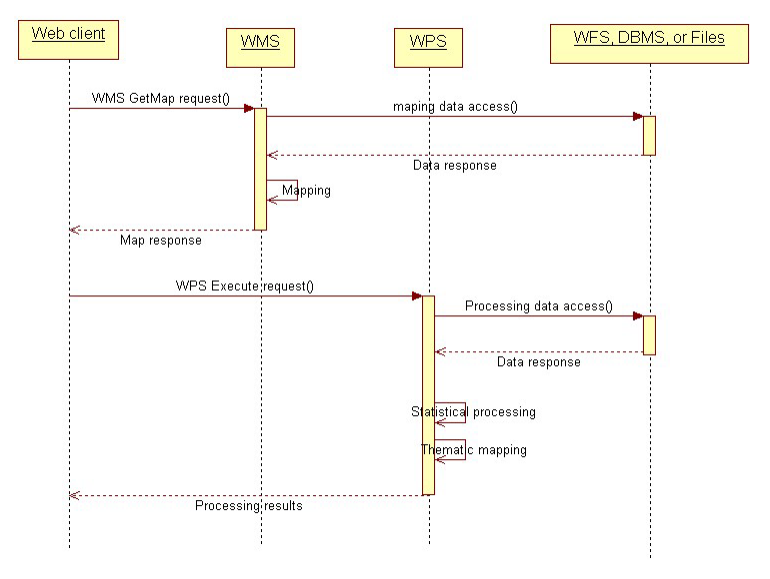
**Service level sequential diagram for health information access**. The Web client invokes the WMS and WPS for health information access. WMS and WPS obtain the raw data from WFS, DBMS, or files, and then perform the mapping and processing operations.

**Figure 10:**
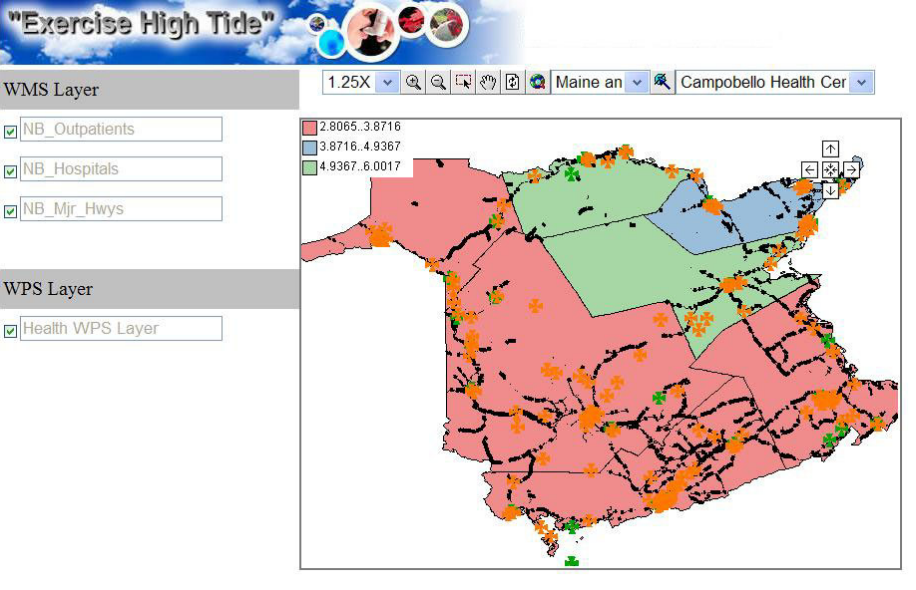
**The exported HTML viewer**. This viewer provides quick access to WMS/WPS services for visualization purposes.

A configuration wizard (See Figure 8) was developed to allow health managers to configure WMS/WPS services for the end users. The number of WMS layers and the parameters of the WPS layer can be set. A sequence diagram of the health information access is shown in Figure 9. After the export process, the generated HTML viewer allows easy and quick access to WMS/WPS services for visualization purposes. As shown in Figure 10, a CMR distribution map from WPS and some facility distribution maps from WMS are integrated. A clinic layer (NB_outpatients) is added as a default layer for locating the clinic locations. It provides users (researchers, health officials, practitioners, policy makers, and epidemiologists) with access to GIS functionality for visualizing health data, and evidence-based decision making on disease outbreaks. The HTML viewer can be saved and used anywhere through the Web, as the JavaScript functions (Zoom in, Pan, etc), WMS services, WPS services are accessed online.

## Discussion

**Figure 11:**
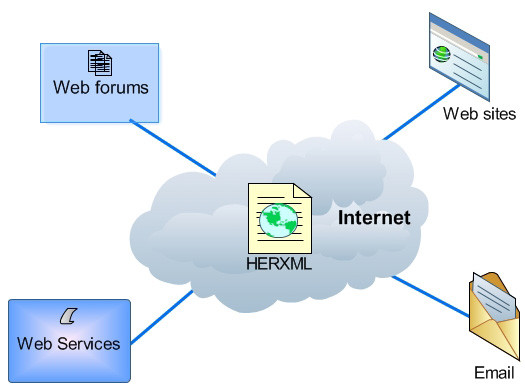
**The sharing of HERXML**. HERXML can be shared via Internet by web forums, web sites, web services, emails, etc.

The HERXML can be used to share the cartographic representation of health information (able to consider a variety of health activities), and describe health data sources and statistical methods. Our implemented HERXML parser can utilize the representation styles in HERXML documents to generate health maps. The HERXML can be shared by users through the Web in many ways such as email, web sites, web forums, and web services regardless of platform or system (See Figure 11). Thus, health information can be easily represented and shared while keeping the secret of raw health information. If the users are interested in the detailed health information, they can contact the data source manager.

Similar to GML and SVG, HERXML is pure XML (using ASCII file) and in a vector format. The ASCII file format makes it easy for humans to read, search, and edit. Although ASCII file format is much larger than the binary format, a number of XML compression techniques methods, namely gZip, XMill, XGrind, Xpress, and XComp, have been developed to improve the performance of transferring XML over the Web [23]. The vector format enables scalability and resolution independence. A file in raster format usually would be much larger in volume than a file in vector format at the same resolution [24]. The HERXML documents can be interpreted as view-only maps (e.g., JPEG) or interactive maps (e.g., SVG) with the attributes and defined representation style in them. Thus, the cartographical representation will be the same in any platform or system.

SVG is designed for computer graphics, and it lacks point feature representation elements and uses inverted y-axis coordinate system, making it unsuited for Web-based cartography [25]. In addition, SVG uses the graphical coordinate, and this leads to some problems in integrating data from different sources together if they do not have the same coordinates. Meanwhile, GML is able to model, transport, and store spatial information, but it can not provide the cartographical representation of spatial information. HERXML utilizes GML in modeling geospatial features and provide point, line, polygon, and chart styles, making it satisfactory in Web-based cartography. Moreover, HERXML integrates many kinds of attributes information together in a well-formatted structure, with the ability to be represented as text, maps and graphics.

Taking advantage of XML, HERXML is extensible, with the potential to add more health information tags to the representation in defining new health parameters or methods. HERXML is simple and well-structured, and the comprehensive description of health data representation will need more extensions. Meanwhile, to improve the semantic meaning in understanding the health data representation, a well defined ontology should be generated to represent shared vocabularies. The development of XML databases, which facilitate the efficient management of XML, will support the storage and manipulation of HERXML documents.

The WPS standard can support both synchronous and asynchronous requests in the execution process. An asynchronous request is very flexible for users in health data processing, especially when the process is computation expensive. During the processing, the dynamically updated execute response document enables users to know the processing status. The results of WPS could use direct data output or a URL which points to the processing results in the server. For health data processing, it is possible for national health organizations to host some processing functions as well as some basic data (e.g., census data) in their server. If a local health organization wants to use the processing and mapping power, they only have to purchase it and make its data accessible to processing servers, and then they can get the processing results conveniently. To reduce the hardware or software investment costs at every local organization, it would be feasible to build a public health infrastructure to support processing power on the Web. In this way, users can flexibly choose the required processing services and assemble them based on their needs. However, regarding the data used in the processing services, the standard method of accessing health data and related data (e.g., temperature data) for web processing still needed to be explored.

## Conclusion

In this research, we developed a HERXML schema to support Web-based representation of health data based on XML specifications, with consideration of semantic, geometric, and graphical aspects of health information. HERXML has been used by the New Brunswick Lung Association in the sharing of health representation information. It provides a suitable way to represent health information for sharing with other users through the Web. The HERXML can be utilized by health practitioners, policy makers, and the public in many areas such as disease etiology, health planning, health resource management, health promotion and health education. The concept definitions and the richness of the vocabularies under the three categories of semantic, geometrical, and cartographical still need to be improved to meet the requirements from the growing users. New application and services has been implemented in CGDI for health surveillance, with many standard WMS, WFS, and WPS services. WPS provides a solution for publishing the health processing and mapping tools online. In our case study, we enabled the online heath data processing and sharing, as well as the reusability and interoperability of health services. The implemented application and services facilitate access to maps and visualization of disease prevalence, mortalities, and determinants of health, transmission patterns, and components of healthcare response. This research brought a new solution in better health data representation and initial exploration of the Web-based processing of health information, and will further promote the growth and enrichment of the CGDI in the public health sector. Our future work will be on the improvement of comprehensiveness of HERXML in health data representation and investigation of data transmission for WPS services.

## Competing interests

The authors declare that they have no competing interests.

## Authors' contributions

All authors participated in the design of the HERXML, and the implementation of the architecture for processing and sharing health information. All authors read and approved the final manuscript.

## Supplementary Material

Additional File 1 **This HERXML schema covers the semantic, geometric and graphic representations of health information**. Click here for file
