# Supplementary material for: Towards Web-based representation and processing of health information
Source: Int J Health Geogr. 2009 Jan 21;8:3. doi: 10.1186/1476-072X-8-3 (PMC2651125; doi:10.1186/1476-072X-8-3)
Supplement: Additional File 1 — This HERXML schema covers the semantic, geometric and graphic representations of health information. [file 1476-072X-8-3-S1.pdf]

```xml
<?xml version="1.0" encoding="UTF-8"?>
<!--
By Sheng Gao, April 2008

Submitted as additional material for the IJHG article

This document is our preliminary HERXML schema, designed using Altova XMLSpy. This XML schema covers the semantic, geometric and graphic representations of health information.
-->

<xs:schema xmlns:herxml="http://nblung.ca" xmlns:xs="http://www.w3.org/2001/XMLSchema" xmlns:gml="http://www.opengis.net/gml" xmlns:xlink="http://www.w3.org/1999/xlink" targetNamespace="http://nblung.ca" elementFormDefault="qualified" attributeFormDefault="unqualified">
    <xs:import namespace="http://www.opengis.net/gml" schemaLocation="schema/gml/3.1.1/base/feature.xsd"/>
    <xs:element name="HERXML">
        <xs:annotation>
            <xs:documentation>herxml schema root element</xs:documentation>
        </xs:annotation>
        <xs:complexType>
            <xs:sequence>
                <xs:element name="Health" type="herxml:HealthType"/>
                <xs:element name="MappingData">
                    <xs:complexType>
                        <xs:sequence>
                            <xs:element ref="herxml:BoundingBox"/>
                            <xs:element name="SpatialData">
                                <xs:complexType>
                                    <xs:sequence>
                                        <xs:element ref="herxml:DataSource"/>
                                        <xs:choice>
                                            <xs:element name="WFS">
                                                <xs:complexType>
                                                    <xs:sequence>
                                                        <xs:element name="URL"/>
                                                        <xs:element name="LayerName"/>
                                                    </xs:sequence>
                                                    <xs:attribute name="version"/>
                                                </xs:complexType>
                                            </xs:element>
                                            <xs:element name="Geometries">
                                                <xs:complexType>
                                                    <xs:sequence>
                                                        <xs:element name="Geometry" type="gml:AbstractGeometricPrimitiveType" maxOccurs="unbounded"/>
                                                    </xs:sequence>
                                                </xs:complexType>
                                            </xs:element>
                                            <xs:element name="RemoteLink">
                                                <xs:complexType>
                                                    <xs:attribute name="type" form="unqualified"/>
                                                    <xs:attribute name="href"/>
                                                </xs:complexType>
                                            </xs:element>
                                        </xs:choice>
                                    </xs:sequence>
                                </xs:complexType>
                            </xs:element>
                            <xs:element name="Relation">
                                <xs:complexType>
                                    <xs:sequence>
                                        <xs:element name="JoinAttribute" type="xs:string"/>
                                        <xs:element name="MatchingValuePairs" maxOccurs="unbounded">
                                            <xs:complexType>
                                                <xs:sequence>
                                                    <xs:element name="SpatialIDValue" type="xs:string"/>
                                                    <xs:element name="HealthIDValue" type="xs:string"/>
                                                </xs:sequence>
                                            </xs:complexType>
                                        </xs:element>
                                    </xs:sequence>
                                </xs:complexType>
                            </xs:element>
                            <xs:element name="MappingValues">
                                <xs:complexType>
                                    <xs:sequence>
                                        <xs:element name="StatisticalMethod">
                                            <xs:complexType>
                                                <xs:sequence>
```

```xml
                          <xs:element ref="herxml:Name"/>
                          <xs:element ref="herxml:Title"/>
                          <xs:element ref="herxml:Description"/>
                          <xs:element name="ParameterGroup" maxOccurs="unbounded">
                            <xs:complexType>
                              <xs:sequence>
                                <xs:element ref="herxml:parameter" maxOccurs="unbounded"/>
                              </xs:sequence>
                            </xs:complexType>
                          </xs:element>
                        </xs:sequence>
                      </xs:complexType>
                    </xs:element>
                    <xs:element ref="herxml:DataSource"/>
                    <xs:element name="MappingValueGroup" maxOccurs="unbounded">
                      <xs:complexType>
                        <xs:sequence>
                          <xs:element name="MappingValue" maxOccurs="unbounded">
                            <xs:complexType>
                              <xs:simpleContent>
                                <xs:extension base="xs:double">
                                  <xs:attribute name="healthIDValue"/>
                                </xs:extension>
                              </xs:simpleContent>
                            </xs:complexType>
                          </xs:element>
                        </xs:sequence>
                        <xs:attribute name="groupAttr"/>
                      </xs:complexType>
                    </xs:element>
                  </xs:sequence>
                  <xs:attribute name="attrName"/>
                </xs:complexType>
              </xs:element>
            </xs:sequence>
          </xs:complexType>
        </xs:element>
        <xs:element name="Representation">
          <xs:complexType>
            <xs:sequence>
              <xs:element ref="herxml:BoundingBox"/>
              <xs:element ref="herxml:Style"/>
            </xs:sequence>
          </xs:complexType>
        </xs:element>
      </xs:sequence>
      <xs:attribute name="version" use="required"/>
    </xs:complexType>
  </xs:element>
  <xs:element name="Name" type="xs:string"/>
  <xs:element name="Title" type="xs:string"/>
  <xs:element name="Description" type="xs:string"/>
  <xs:element name="KeywordList">
    <xs:complexType>
      <xs:sequence>
        <xs:element name="Keyword" type="xs:string" maxOccurs="unbounded"/>
      </xs:sequence>
    </xs:complexType>
  </xs:element>
  <xs:element name="BoundingBox" type="herxml:BoundingBoxType"/>
  <xs:complexType name="BoundingBoxType">
    <xs:sequence>
      <xs:element name="Minx" type="xs:double"/>
      <xs:element name="MinY" type="xs:double"/>
      <xs:element name="MaxX" type="xs:double"/>
      <xs:element name="MaxY" type="xs:double"/>
    </xs:sequence>
    <xs:attribute name="srsName" type="xs:string" use="required"/>
  </xs:complexType>
  <xs:element name="parameter" type="xs:string" abstract="true">
    <xs:annotation>
      <xs:documentation>abstract parameter element, used for the definition  of health infulential factors</xs:documentation>
    </xs:annotation>
  </xs:element>
  <xs:element name="Geolayer" type="xs:string" substitutionGroup="herxml:parameter"/>
  <xs:element name="AgeFrom" type="xs:string" substitutionGroup="herxml:parameter"/>
```

```xml
    <xs:element name="AgeTo" type="xs:string" substitutionGroup="herxml:parameter"/>
    <xs:element name="StartTime" type="xs:string" substitutionGroup="herxml:parameter"/>
    <xs:element name="EndTime" type="xs:string" substitutionGroup="herxml:parameter"/>
    <xs:element name="Gender" type="xs:string" substitutionGroup="herxml:parameter"/>
    <xs:complexType name="StyleType">
        <xs:annotation>
            <xs:documentation>abstract representation style, used for the defintion of PointStyleType, ChartStyleType, LineStyleType, and PolygonStyleType</xs:documentation>
        </xs:annotation>
        <xs:sequence>
            <xs:element ref="herxml:Name" minOccurs="0"/>
            <xs:element ref="herxml:Title" minOccurs="0"/>
            <xs:element ref="herxml:Description" minOccurs="0"/>
        </xs:sequence>
    </xs:complexType>
    <xs:element name="Style" type="herxml:StyleType" abstract="true"/>
    <xs:element name="PointStyle" type="herxml:PointStyleType" substitutionGroup="herxml:Style"/>
    <xs:element name="ChartStyle" type="herxml:ChartStyleType" substitutionGroup="herxml:Style"/>
    <xs:element name="LineStyle" type="herxml:LineStyleType" substitutionGroup="herxml:Style"/>
    <xs:element name="PolygonStyle" type="herxml:PolygonStyleType" substitutionGroup="herxml:Style"/>
    <xs:complexType name="PointStyleType">
        <xs:complexContent>
            <xs:extension base="herxml:StyleType">
                <xs:sequence>
                    <xs:element name="PointSize" type="xs:double"/>
                    <xs:choice>
                        <xs:element ref="herxml:Color"/>
                        <xs:element name="Symbol">
                            <xs:complexType>
                                <xs:sequence>
                                    <xs:element ref="herxml:Name"/>
                                    <xs:element name="URL" type="xs:string"/>
                                </xs:sequence>
                            </xs:complexType>
                        </xs:element>
                    </xs:choice>
                </xs:sequence>
            </xs:extension>
        </xs:complexContent>
    </xs:complexType>
    <xs:complexType name="LineStyleType">
        <xs:complexContent>
            <xs:extension base="herxml:StyleType">
                <xs:sequence>
                    <xs:element ref="herxml:Color"/>
                    <xs:element name="LineWeight" type="xs:double"/>
                    <xs:element name="LineStyle" type="xs:string"/>
                </xs:sequence>
            </xs:extension>
        </xs:complexContent>
    </xs:complexType>
    <xs:complexType name="ChartStyleType">
        <xs:complexContent>
            <xs:extension base="herxml:StyleType">
                <xs:sequence>
                    <xs:element name="ChartMethod" type="xs:string"/>
                    <xs:element name="ChartSize" type="xs:double"/>
                    <xs:element name="ChartVariation" type="xs:boolean"/>
                    <xs:element name="ChartColorScheme" maxOccurs="unbounded">
                        <xs:complexType>
                            <xs:sequence>
                                <xs:element name="ChartField" type="xs:string"/>
                                <xs:element ref="herxml:Color"/>
                            </xs:sequence>
                        </xs:complexType>
                    </xs:element>
                </xs:sequence>
            </xs:extension>
        </xs:complexContent>
    </xs:complexType>
    <xs:complexType name="PolygonStyleType">
        <xs:complexContent>
            <xs:extension base="herxml:StyleType">
                <xs:sequence>
                    <xs:element ref="herxml:Fill"/>
                    <xs:element name="Border" type="herxml:LineStyleType"/>
```

```xml
      </xs:sequence>
    </xs:extension>
  </xs:complexContent>
</xs:complexType>
<xs:complexType name="FillType">
  <xs:sequence>
    <xs:element ref="herxml:Description" minOccurs="0"/>
  </xs:sequence>
</xs:complexType>
<xs:element name="Fill" type="herxml:FillType"/>
<xs:element name="RangeFill" type="herxml:RangeFillType" substitutionGroup="herxml:Fill"/>
<xs:element name="GradientFill" type="herxml:GradientFillType" substitutionGroup="herxml:Fill"/>
<xs:complexType name="GradientFillType">
  <xs:complexContent>
    <xs:extension base="herxml:FillType">
      <xs:sequence>
        <xs:element name="FromColor" type="xs:string"/>
        <xs:element name="ToColor" type="xs:string"/>
        <xs:element name="NumOfClasses" type="xs:int"/>
      </xs:sequence>
    </xs:extension>
  </xs:complexContent>
</xs:complexType>
<xs:complexType name="RangeFillType">
  <xs:complexContent>
    <xs:extension base="herxml:FillType">
      <xs:choice>
        <xs:element name="SingleRange" maxOccurs="unbounded">
          <xs:complexType>
            <xs:sequence>
              <xs:element name="RangeValue" type="xs:double"/>
              <xs:element ref="herxml:FillMethod"/>
            </xs:sequence>
          </xs:complexType>
        </xs:element>
        <xs:element name="DoubleRange" maxOccurs="unbounded">
          <xs:complexType>
            <xs:sequence>
              <xs:element name="MinValue" type="xs:double"/>
              <xs:element name="MaxValue" type="xs:double"/>
              <xs:element ref="herxml:FillMethod"/>
            </xs:sequence>
          </xs:complexType>
        </xs:element>
      </xs:choice>
    </xs:extension>
  </xs:complexContent>
</xs:complexType>
<xs:element name="Color" type="xs:string">
  <xs:annotation>
    <xs:documentation>e.g., 0x######</xs:documentation>
  </xs:annotation>
</xs:element>
<xs:element name="Texure"/>
<xs:element name="Pattern"/>
<xs:complexType name="FillMethodType">
  <xs:choice>
    <xs:element ref="herxml:Color"/>
    <xs:element ref="herxml:Texure"/>
    <xs:element ref="herxml:Pattern"/>
  </xs:choice>
</xs:complexType>
<xs:element name="FillMethod" type="herxml:FillMethodType"/>
<xs:complexType name="DataSourceType">
  <xs:sequence>
    <xs:element name="Contact">
      <xs:complexType>
        <xs:sequence>
          <xs:element name="ContactName" type="xs:string"/>
          <xs:element name="Address" type="xs:string"/>
          <xs:element name="Phone" type="xs:string"/>
        </xs:sequence>
      </xs:complexType>
    </xs:element>
    <xs:element name="DataSourceDescription" type="xs:string"/>
    <xs:element name="DataSourceTime" type="xs:string"/>
```

```xml
		</xs:sequence>
	</xs:complexType>
	<xs:element name="DataSource" type="herxml:DataSourceType"/>
	<xs:complexType name="HealthType" abstract="true">
		<xs:sequence>
			<xs:element ref="herxml:Name"/>
			<xs:element ref="herxml:Title"/>
			<xs:element ref="herxml:Description"/>
			<xs:element ref="herxml:KeywordList" minOccurs="0"/>
		</xs:sequence>
		<xs:attribute name="type" type="xs:string" use="optional"/>
	</xs:complexType>
	<xs:complexType name="DiseaseObservationType">
		<xs:complexContent>
			<xs:extension base="herxml:HealthType">
				<xs:sequence>
					<xs:element name="Code" type="xs:string"/>
				</xs:sequence>
			</xs:extension>
		</xs:complexContent>
	</xs:complexType>
</xs:schema>
```
